# The Frozen Elephant Trunk Procedure—8 Years of Experience from Poland

**DOI:** 10.3390/jcm13216544

**Published:** 2024-10-31

**Authors:** Marian Burysz, Grzegorz Horosin, Wojciech Olejek, Mariusz Kowalewski, Krzysztof Bartuś, Artur Słomka, Radosław Litwinowicz, Jakub Batko

**Affiliations:** 1 Department of Cardiac Surgery, Regional Specialist Hospital, 86-300 Grudziądz, Poland; 2 Thoracic Research Centre, Collegium Medicum Nicolaus Copernicus University, Innovative Medical Forum, 85-094 Bydgoszcz, Poland; 3 CAROL—Cardiothoracic Anatomy Research Operative Lab, Department of Cardiovascular Surgery and Transplantology, Institute of Cardiology, Jagiellonian University Medical College, 31-008 Krakow, Poland; 4 Department of Cardiac Surgery, Centre of Post-Graduate Medical Education, Central Clinical Hospital of the Ministry of Interior, 01-813 Warsaw, Poland; 5 Department of Cardiovascular Surgery and Transplantology, Institute of Cardiology, Jagiellonian University Medical College, 31-008 Krakow, Poland; 6 Department of Pathophysiology, Ludwik Rydygier Collegium Medicum in Bydgoszcz, Nicolaus Copernicus University in Toruń, 85-094 Bydgoszcz, Poland; artur.slomka@cm.umk.pl; 7 Department of Anatomy, Jagiellonian University Medical College, 31-008 Krakow, Poland

**Keywords:** aortic aneurysm, aortic arch repair, frozen elephant trunk, hybrid cardiac surgery, Thoraflex

## Abstract

**Background:** The frozen elephant trunk method combines the implantation of a Dacron prosthesis with a self-expanding stent graft, which allows for complex repairs of the aortic arch and thoracic aorta in one procedure. Despite the advantages of hybrid treatment for aortic arch aneurysms, in Poland, only a few such surgeries are performed annually compared to in Western countries. The aim of this study was to demonstrate the 8-year outcomes of treatment at the center where the Aortic Team operates, which is one of the centers in Poland with the most extensive experience in hybrid FET treatment. **Methods:** Patients who underwent frozen elephant trunk surgery for chronic and acute pathologies of the aortic arch and thoracic aorta between March 2016 and March 2024 were comprehensively analyzed retrospectively. Frozen elephant trunk procedures were performed under three consecutive clinical conditions: acute aortic dissection, chronic aortic dissection and redo surgery. **Results:** A total of 40 patients (median age: 60 years (53–66), 67.5% male) were admitted to our hospital and underwent an FET procedure. The median Euroscore II was 25.9% and the 30-day mortality was 7.5%. The 1-year and 5-year mortalities were the same, equal to 15%, with mortality cases observed only in the first and second groups of consecutive patients during the first two months of follow-up. Spinal cord injury was observed in 2.5% of patients. **Conclusions:** The FET technique can be successfully used to treat aortic aneurysms with optimal results and low complication rates. The surgery length, including the cardiopulmonary bypass and aortic cross-clamp times, decreased significantly with increasing experience.

## 1. Introduction

Acute aortic dissection remains a life-threatening condition and a major challenge for cardiac surgeons. Its incidence is estimated at six new cases per 100 000 population per year [1]. The current American Heart Association guidelines emphasize the urgency of surgical intervention or thoracic endovascular aortic repair for acute aortic syndrome [2]. It is promising that in recent years a growing number of centers have succeeded at reducing the number of complications associated with complex aortic arch surgery and implementing more patient-tailored solutions. One of the most significant advances in this matter has been the reduction of the procedure to a single step, which has been achieved through the introduction of the frozen elephant trunk (FET) method.

**Figure 1:**
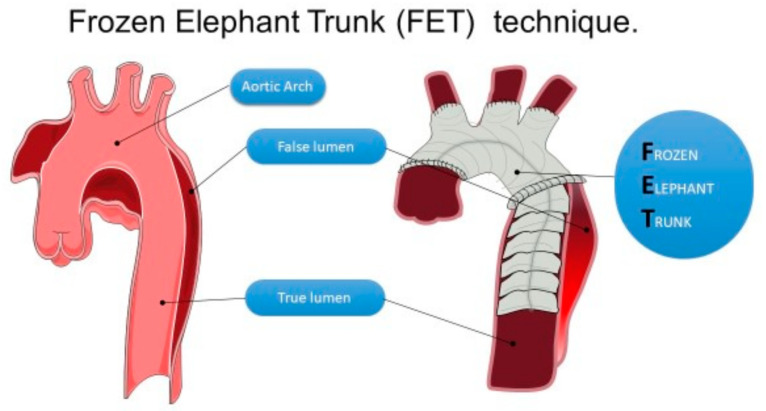
The frozen elephant trunk technique.

The FET method was introduced and described by Karck in 2003 as a modification of the conventional elephant trunk procedure [3]. It combines the implantation of a Dacron prosthesis with a self-expanding stent graft that is sutured to its landing zone (frozen trunk) (Figure 1).

With this approach, complex repairs of the aortic arch and thoracic aorta can be performed in one operation so that patients are not exposed to the risk of a necessary second procedure [3]. In addition, the operation time is shortened, the time of extracorporeal circulation is reduced and proximal endoleaks and stent graft displacement are prevented [4]. In this context, FET has been shown to significantly improve the patient prognosis compared to the conventional elephant trunk approach [4,5]. This approach is particularly beneficial for patients with aortic disease extending from the aortic arch to the supra-arch vessels. While FET remains a major breakthrough in cardiothoracic surgery, ongoing discussions in the literature focus on the prevention of spinal cord ischemia (SCI) and the search for the most optimal surgical approach, including the level of hypothermia, cerebral perfusion strategies and reductions in the extracorporeal circulation time [6].

Unfortunately, despite the advantages of hybrid treatment for aortic arch aneurysms, only a few such surgeries are performed in Poland compared to in Western countries due to its lower experience in hybrid treatment methods [7,8].

The aim of this study was to demonstrate the 8-year outcomes of treatment at the center where the Aortic Team operates, which is one of the centers in Poland with the most extensive experience in hybrid FET treatment, incorporating technical specifications, comprehensive patient characteristics and postoperative mortality and complication profiles. The results of total aortic arch replacement with the FET procedure were evaluated in patients with aneurysms and/or dissections of the aortic arch and thoracic aorta, including redo operations.

## 2. Materials and Methods

### 2.1. Patients’ Characteristics and Definitions

All patients who underwent FET surgery for chronic and acute pathologies of the aortic arch and thoracic aorta between 1 March 2016 and 31 March 2024 at the Regional Specialized Hospital in Grudziadz, Poland, were comprehensively analyzed retrospectively. Patients were mostly operated on by the first author (35/40, 87.5%) but, in three cases, the first author was assisted in the procedure by a specialized team of two anesthetists. FET procedures were performed under three consecutive clinical conditions, defined below, according to guidelines [2]:

Acute aortic dissection (AAD)—patients with the onset of symptoms < 14 days from surgery;
Chronic aortic dissection (non-AAD)—patients with the onset of symptoms > 14 days from surgery;
Redo surgery—patients with previous repair/surgery in any part of the thoracic aorta.

The two prostheses available in Europe were used, namely, the Thoraflex Hybrid prosthesis (Terumo Aortic, Vascutek Ltd., Inchinnan, UK) and the E-vita Neo Open (JOTEC GmbH, Hechingen, Germany). The choice of the stent graft size was determined based on the aortic dimensions and the circumference of the affected region made using virtual calipers on a computed tomography angiography of the aorta. 

Preoperative characteristics and complications were collected and defined accordingly based on patients’ documentation notifications or them meeting the clinical requirements of each disease [2].

Due to the retrospective nature of this study, the approval of the Bioethics Committee was waived by the local medical council’s Bioethics Committee. The study protocol complies with the ethical guidelines of the Declaration of Helsinki of 1975.

### 2.2. Surgical Management

All procedures were performed via median sternotomy under general anesthesia and hypothermia with temperatures between 20 and 32 degrees Celsius (°C). Carbon dioxide (CO_2_) flooding of the surgical field was employed in all cases to mitigate the risk of air embolism. Each patient underwent cardiopulmonary bypass (CBP) using a centrifugal pump with hemofiltration, while intraoperative cell salvage was used selectively. An arterial cannula for the CBP was placed in the right or left femoral artery whenever feasible; alternatively, cannulation was conducted through the left subclavian and brachiocephalic arteries in other cases. Cerebral perfusion was maintained by the bilateral antegrade strategy. The procedure consisted in repairing the proximal aortic arch and deploying a distal stent graft, combined with the Bentall procedure, the Florida sheath or additional stent grafts if necessary. The prostheses utilized measured 100 or 150 mm for the Thoraflex devices and 120 mm for the E-vita Neo Open. In cases requiring stent graft elongation, this step was undertaken accordingly. Delayed chest closure was performed if deemed necessary based on the patient’s condition. The stent graft was positioned in Ishimura landing zone 2 or 4, reinforced with a felt strip and then inserted into the distal part of the thoracic aorta with a previously placed guidewire. Following the selection and adjustment of the length, the prostheses were anastomosed with the sinotubular junction/artificial valve. Then, the supra-arch vessels were reconnected, starting with the left subclavian artery, followed by the left common carotid artery and finally the brachiocephalic trunk.

### 2.3. Data Management and Endpoints

Demographic data, preoperative comorbidities and preoperative imaging findings were systematically extracted from both conventional and electronic medical records. Intraoperative details were meticulously recorded from the surgical reports. In addition, postoperative outcomes such as the 30-day mortality, complications, the length of hospital stay and the need for next-stage surgery were meticulously documented. The 1-year and 5-year mortality rates were collected from the National Health Fund, an obligatory, public health insurance institution in Poland, incorporated into the KROK (Polish National Registry of Cardiac Surgery Procedures) registry (available at: www.krok.csioz.gov.pl, accessed on 10 May 2024). Additionally, patients were stratified into four equal groups based on their admission times to determine differences in the outcomes with increasing center experience. 

### 2.4. Statistical Analysis

Data were analyzed using IBM SPSS Statistics 29.0 (Predictive Solutions, Pittsburgh, PA, USA). Categorical variables are presented as numbers (n) or percentages. Quantitative variables are presented as medians with first and third quartiles. The normal distribution was analyzed using the Shapiro–Wilk test. Multi-group continuous variable differences were assessed using the Kruskal and Wallis test with Dunn’s post hoc test with Bonferroni correction if the results of the Kruskal and Wallis test were statistically significant. For categorical variables, the chi-square test for independence or Fischer’s exact test was used. For the operative time, cardiopulmonary bypass time, aortic cross-clamp time and distal hypothermic circulatory arrest time, learning curves with confidence intervals were performed. One-year and five-year survival curves were performed for all of the patients with division into four equal consecutive subgroups. In subgroups 1 and 2, 5-year follow-up was performed in all of the patients. In subgroup 3, the median follow-up time was 2.4 (1.9–3.4) years. In subgroup 4, the median follow-up time was 0.6 (0.4–1.5) years. A *p*-value < 0.05 was considered statistically significant.

## 3. Results

### 3.1. Characteristics of the Patients

Between March 2016 and March 2024, 40 patients (median age: 60 years (53–66), 67.5% male) were admitted to our hospital and underwent an FET procedure. Detailed patient characteristics are presented in Table 1. 

Significant differences were observed between the groups at baseline based on the indication for surgery regarding the choice of surgery, atrial fibrillation rate, smoking status, affected aortic segments and prior aortic procedures. In the consecutive patient groups, preoperative differences were noted in the pulmonary disease rate and preoperative ischemic complications (see Supplementary Table S1).

### 3.2. Intraoperative and Postoperative Outcomes

There were no statistical differences in the intraoperative and postoperative data between the groups determined by surgical indication, with the exception of the anastomosis zone. Detailed data can be found in Table 2 and Table 3.

Statistically significant differences were observed in the intraoperative and postoperative outcomes between the patient groups, including the 30-day mortality (deaths were observed only in the second group, *p* = 0.02), hospitalization time (shortest in the second group, *p* = 0.049), operative time (shortest in the fourth group, with a median value twice as high as in the first group, *p* < 0.001), cardiopulmonary bypass time (shortest in the fourth group, twice as high as in the first group, *p* < 0.001), aortic cross-clamp time (shortest in the fourth group, *p* = 0.03) and distal hypothermic circulatory arrest time (similar in the second and fourth groups, *p* = 0.03). The 1-year and 5-year mortalities were the same, equal to 15%, with mortality cases observed only in the first and second groups of consecutive patients during the first two months of follow-up. 

Detailed exact comparisons between the intraoperative characteristics can be found in Table 4. 

**Figure 2:**
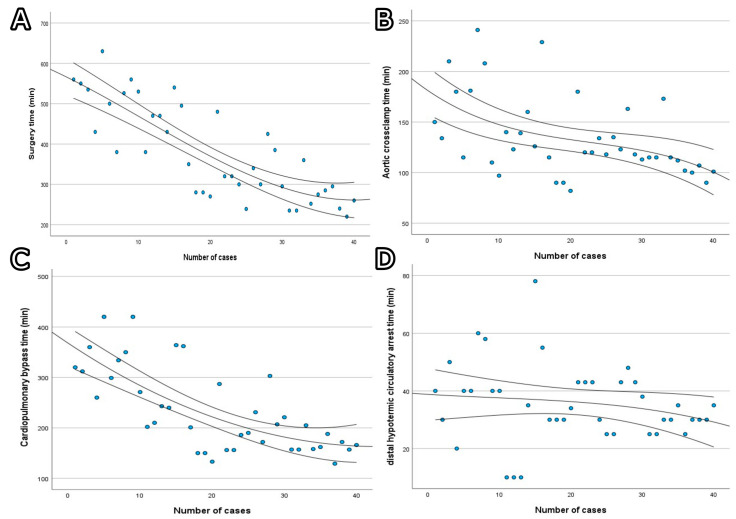
Learning curves with confidence intervals: (**A**) surgery time; (**B**) cardiopulmonary bypass time; (**C**) aortic cross-clamp time; (**D**) distal hypothermic circulatory arrest time.

The learning curves for the operative time, cardiopulmonary bypass time, aortic cross-clamp time and distal hypothermic circulatory arrest time can be found in Figure 2A–D. 

**Figure 3:**
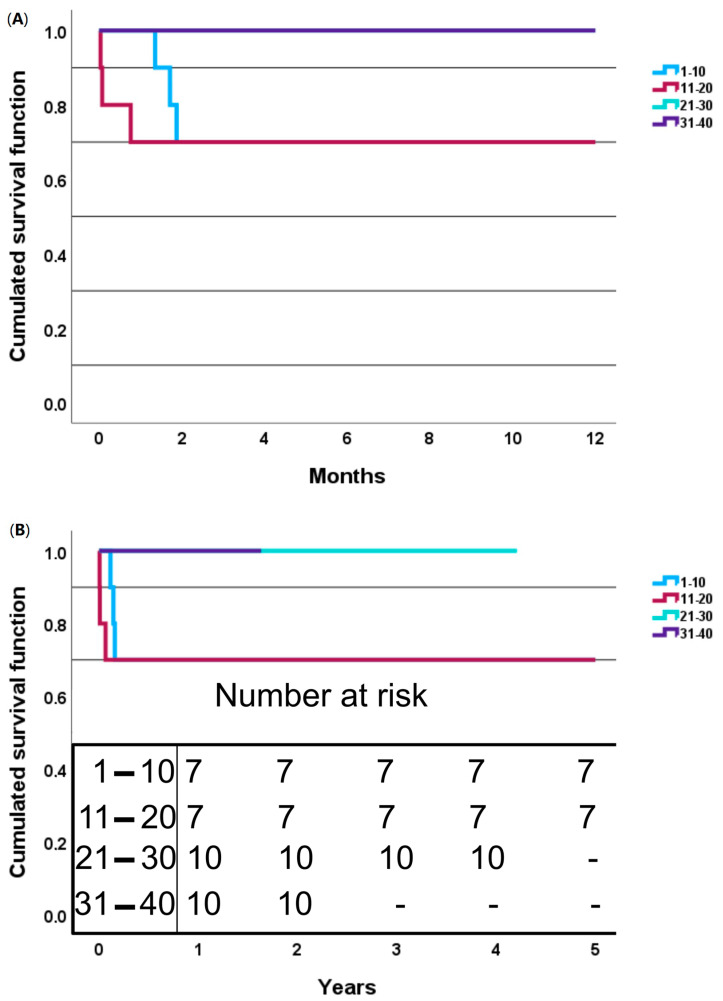
Survival curves with division into four consecutive groups of patients: (**A**)—one-year survival curves; (**B**)—five-year survival curves.

Detailed survival curves can be found in Figure 3A,B. 

Detailed comparisons between the postoperative characteristics can be found in Supplementary Table S2.

## 4. Discussion

Our approach to the FET procedure can be generally identified with the use of hybrid devices and antegrade bilateral cerebral perfusion with moderate hypothermia. Additionally, cannulation via the femoral artery was preferred in our hospital, with 92.5% without any complications. Considering the extensive and demanding nature of FET, a 30-day mortality rate of 7.5% should be considered excellent given a median Euroscore II of 25.9%. It is worth noting that in the 8 years that we have been performing FET, not a single case of 30-day mortality after FET has been observed at our center after the third year of using this procedure, which not only serves as evidence of the complexity of the technique but also underscores the potential for excellent outcomes with increasing experience within the surgical department. At our center, in most cases, delayed chest closure with packing is utilized as a modification proposed by the main surgeon due to patients’ preoperative anticoagulation and hemostasis dysfunction, which provides good results in terms of in-hospital complications and mortality. It is worth noting that most of the procedures were performed by one surgeon with a specialized anesthesia team, which may have played a crucial role in the gradual improvement during the procedures.

We opted for the “proximal” route when applying the FET and fixed it in zone 2 whenever possible. Recent studies show that a distal anastomosis in zone 2 leads to better outcomes in terms of long-term mortality and an improvement in the 5-year survival rate. Choudhury et al. found in their analysis that FET in zone 2 has a lower incidence of SCI, renal complications and respiratory failure compared to in zone 3 [9]. In another single-center study, Arnold et al. observed beneficial outcomes when zone 2 was chosen as the target proximal fixation site, such as better exposure with improved hemostasis and a lower risk of left pharyngeal nerve injury. It should be known that, in certain clinical situations, distal anastomosis can be performed in zone 4. In our practice, such an approach was performed in patients with unfavorable tissue conditions in zone 2. Arnold et al. reported an SCI rate of 2.7%; however, they defined SCI exclusively as permanent paraparesis or paraplegia and included not only total but also partial arch repairs in their patients [10]. The prevalence of postoperative SCI in our study group was 2.5%. According to the available studies, the incidence of SCI ranges from 0.8 to 8.9% [11,12,13]. SCI has always been considered an inherent risk of FET. Nevertheless, FET has gained popularity and is increasingly seen as advantageous compared to the classic elephant trunk technique, mainly due to its lower early mortality, better overall survival rate and single-stage nature [4]. Currently, SCIs are consensually regarded as multifactorial events requiring comprehensive prophylaxis and treatment [12]. New-onset cerebral ischemia was observed in three patients (7.5%) in our study, while the total number of postoperative cerebral ischemia cases was five (12.5%). Shen et al. presented their approach for acute type I aortic dissection under mild hypothermia (≥30 °C) in 2022 [6]. They achieved promising results and low 30-day mortality (9.8% of all patients) by modifying the sequence of anastomoses (starting with proximal aorta/root reconstruction followed by LCCA management) and the brain-heart-first strategy (“CBP”) technique. However, the patients in their study were significantly younger and less likely to have hypertension compared to our patients. It should be noted that mild hypothermia is suggested for circulatory arrest times under 15 min to prevent cerebral complications. In our study, such complications were observed only in patients with AAD and, without its presence, in patients undergoing non-AAD or redo FET operations. Despite the use of mild hypothermia, which was chosen due to personal experience and the suggestion of the operator, only three patients (7.5%) had new-onset cerebral ischemia, which is comparable to previously published studies, as discussed above. Nevertheless, FET modifications based on debranching and the search for optimal temperatures are more frequently mentioned and discussed in the literature. The available meta-analyses and reviews indicate that the 30-day mortality after FET ranges from 0% to 18.2% [4,14,15], which is comparable to the 7.5% in our study. Malvindi et al. [16] described their interesting approach based on normothermia and no systemic circulatory arrest. A retrogradely inserted stent graft with the cannulation of the brachiocephalic and femoral arteries for CBP, followed by the occlusion of the stent with an endoballoon, allowed them to maintain systemic perfusion at 34–35 °C. Brain protection was achieved by antegrade selective bilateral cerebral perfusion. In this way, they were able to anastomose the graft to the stent without the need for hypothermia or systemic circulatory arrest. Their study used a more conventional FET method with a separate endovascular graft and prosthesis. Out of a total of 13 patients, most with chronic arch aneurysms, they reported two postoperative acute kidney injuries, one in-hospital death due to pulmonary complications and no SCI [16]. 

One of the postoperative complications of FET is stent graft-induced new entry (SINE). This can be defined as any newly detected connection between false and true lumens at the distal or proximal end of the stent graft not observed during the procedure or in the final imaging after the procedure. It can be divided into two types: proximal, in most cases associated with retrograde reperfusion or new retrograde type A dissection, and distal, leading to antegrade dissection or false lumen reperfusion. We did not observe such complications in our population.

Another highlighted consequence of FET is kinking. Although rare, with a prevalence of 0 to 8%, it can lead to life-threatening complications, including septic embolism and multiorgan ischemia due to the presumed formation of an intraluminal thrombus. The identification of kinks can occur both intraoperatively and postoperatively [6,15,17]. In our cohort of 35 patients, we did not observe any cases of kink formation, either during surgery or in the subsequent imaging studies during the postoperative and annual follow-ups. Kayali et al. [14,15] listed three possible causes of buckling. The first is the presence of retrograde blood flow through the FET prosthesis, including retrograde cerebral perfusion, leading to kinking and the deployment of the distal graft in the descending thoracic aorta due to blood accumulation. Secondly, a correlation between longer prostheses and the occurrence of kinking has been suggested, with the ideal length being in the range of 7–8 cm. However, this is not a definitive and clear factor, as there are studies based on longer prostheses in which no cases of buckling were reported. No kinking was found in this study either, so it is another counterargument for the second hypothesis. Finally, the positioning of the stent graft at the flexure of the aorta is thought to increase the risk of kinking at the site of the anastomosis between the stented and unstented portions of the graft. However, the Japanese FET device Frozenix has been reported. Thoraflex and E-vita hybrid prostheses do not contain non-stented segments; therefore, this does not apply to them [14,15]. The current knowledge does not clearly indicate which approach is more beneficial. We believe that this approach is most beneficial in patients with lower-extremity ischemia, as this extremity should be chosen as the preferred site for cannulation. A precise evaluation to obtain the technique best tailored to the patient has yet to be performed. In this study, we show our methods and results based exclusively on hybrid devices and antegrade bilateral cerebral perfusion.

### Limitations

Some limitations to the performed study should be mentioned for a clear interpretation of the results. The presented study was a single-center, retrospective study, with follow-up limited to a 5-year period. Additionally, only 40 patients were included; however, it should be mentioned that all of the patients operated on at our center with this method were included in this study. Additionally, most of the patients were operated on for aortic dissection.

## 5. Conclusions

The FET technique can be successfully used to treat aortic dissection with optimal results and low complication rates. The surgery length, including the cardiopulmonary bypass and aortic cross-clamp times, decreased significantly with increasing experience.

## Figures and Tables

**Table 1:** Patients’ baseline characteristics.

		AAD (n = 28)	Non-AAD (n = 6)	Redo FET (n = 6)	Total	*p*
Age (years)		60 (53–67)	64 (60–65)	51 (42–64)	60 (53–66)	0.34
Male		18 (64.3%)	4 (66.7%)	5 (83.3%)	27 (67.5%)	0.66
Body mass index (kg/m^2^)		28.7 (24.1–30.8)	24.1 (23.4–27.1)	27.2 (21–30.7)	27.8 (23.4–30.7)	0.59
DM2		3 (10.7%)	0 (0%)	0 (0%)	3 (7.5%)	0.50
Hypertension		26 (92.9%)	6 (100%)	5 (83.3%)	37 (92.5%)	0.54
Atrial fibrillation		0 (0%)	0 (0%)	2 (33.3%)	2 (5%)	**0.003**
Chronic heart failure		1 (3.6%)	0 (0%)	0 (0%)	1 (2.5%)	0.80
Pulmonary disease		7 (25%)	1 (16.7%)	3 (50%)	11 (27.5%)	0.37
Ever smoker	Active	20 (71.4%)	0 (0%)	2 (33.3%)	22 (55%)	**0.02**
	Previous	7 (25%)	5 (83.3%)	3 (50%)	15 (37.5%)	
Vascular disease	Peripheral	8 (28.6%)	2 (33.3%)	3 (50%)	13 (32.5%)	0.25
	Cerebral	0 (0%)	1 (16.7%)	0 (0%)	1 (2.5%)	
	Both	3 (10.7%)	0 (0%)	0 (0%)	3 (7.5%)	
Non-elective surgery		27 (96.4%)	3 (50%)	1 (16.7%)	31 (77.5%)	**<0.001**
Tamponade		5 (17.9%)	1 (16.7%)	1 (16.7%)	7 (17.5%)	0.99
Ejection fraction (%)		40 (40–48)	60 (40–65)	45 (40–50)	40 (40–53)	0.18
Euroscore II		29.5 (22.8–48.1)	23.6 (14.3–28.6)	8 (5.5–11.6)	25.9 (11.7–38.7)	0.05
Parts of aorta involved	Type A	8 (28.6%)	0 (0%)	0 (0%)	8 (20%)	**0.007**
	Ascending + arch	12 (42.9%)	2 (33.3%)	3 (50%)	17 (42.5%)	
	Ascending + arch + descending	3 (10.7%)	2 (33.3%)	0 (0%)	5 (12.5%)	
	Arch + descending	0 (0%)	2 (33.3%)	3 (50%)	5 (12.5%)	
	Thoracic + abdominal	5 (17.9%)	0 (0%)	0 (0%)	5 (12.5%)	
Preoperative new-onset neurological symptoms	TIA	9 (32.1%)	1 (16.7%)	2 (33.3%)	12 (30%)	0.51
	Peripheral paresis	3 (10.7%)	0 (0%)	0 (0%)	3 (7.5%)	
	Cerebral ischemia	4 (14.3%)	0 (0%)	0 (0%)	4 (10%)	
Preoperative ischemic non-neurological symptoms	Lower-limb ischemia	10 (35.7%)	1 (16.7%)	2 (33.3%)	13 (32.5%)	0.39
	Cardiac arrest	4 (14.3%)	2 (33.3%)	0 (0%)	6 (15%)	
	Lower-limb ischemia and cardiac arrest	3 (10.7%)	0 (0%)	0 (0%)	3 (7.5%)	
	Mesenteric ischemia	1 (3.6%)	0 (0%)	0 (0%)	1 (2.5%)	
	Mesenteric and lower-limb ischemia	0 (0%)	0 (0%)	1 (16.7%)	1 (2.5%)	
Previous aortic procedures	AVR	0 (0%)	0 (0%)	2 (33.3%)	2 (5%)	**<0.001**
	Abdominal stent graft	2 (7.1%)	2 (33.3%)	0 (0%)	4 (10%)	
	AAD surgery	0 (0%)	1 (16.7%)	2 (33.3%)	3 (7.5%)	
	Bentall procedure	0 (0%)	0 (0%)	2 (33.3%)	2 (5%)	

AAD—acute aortic dissection; AVR—aortic valve replacement; DM2—diabetes mellitus type 2; FET—frozen elephant trunk; TIA—transient ischemic attack. Significant *p* value bolded.

**Table 2:** Intraoperative characteristics.

		AAD (n = 28)	Non-AAD (n = 6)	Redo FET (n = 6)	Total	*p*
Hypothermia temperature (°C)		28 (28–32)	29 (28–32)	28 (28–28)	28 (28–32)	0.80
Surgery time (min)		320 (280–488)	465 (380–560)	350 (270–385)	355 (280–488)	0.25
Cardiopulmonary bypass time (min)		195 (158–293)	323 (202–420)	206 (157–210)	206 (160–301)	0.07
Aortic cross-clamp time (min)		120 (110–162)	126 (115–140)	121 (90–135)	120 (111–155)	0.71
Distal hypothermic circulatory arrest time (min)		35 (30–43)	35 (25–40)	30 (25–34)	35 (30–43)	0.40
Type of surgery	FET	21 (75%)	4 (66.7%)	6 (100%)	31 (77.5%)	0.53
	FET + Bentall	2 (7.1%)	0 (0%)	0 (0%)	2 (5%)	
	FET + Florida sleeve	3 (10.7%)	2 (33.3%)	0 (0%)	5 (12.5%)	
	Complex procedure	2 (7.1%)	0 (0%)	0 (0%)	2 (5%)	
Prosthesis length	100	12 (42.9%)	1 (16.7%)	5 (83.3%)	18 (45%)	0.16
	120	2 (7.1%)	0 (0%)	0 (0%)	2 (5%)	
	150	14 (50%)	5 (83.3%)	1 (16.7%)	20 (50%)	
Arterial cannulation site	Femoral	26 (92.9%)	5 (83.3%)	6 (100%)	37 (92.5%)	0.63
	Brachiocephalic	1 (3.6%)	1 (16.7%)	0 (0%)	2 (5%)	
	Subclavian	1 (3.6%)	0 (0%)	0 (0%)	1 (2.5%)	
Anastomosis zone	1	2 (7.1%)	0 (0%)	0 (0%)	2 (5%)	0.03
	2	22 (78.6%)	2 (33.3%)	6 (100%)	30 (75%)	
	4	4 (14.3%)	4 (66.7%)	0 (0%)	8 (20%)	
Cell server		17 (60.7%)	2 (33.3%)	4 (66.7%)	23 (57.5%)	0.42
Delayed chest closure		25 (89.3%)	6 (100%)	5 (83.3%)	36 (90%)	0.61

AAD—acute aortic dissection; FET—frozen elephant trunk.

**Table 3:** Postoperative characteristics and complication rates.

		AAD (n = 28)	Non-AAD (n = 6)	Redo FET (n = 6)	Total	*p*
30-day mortality		2 (7.1%)	0 (0%)	1 (16.7%)	3 (7.5%)	0.54
Complications	Peripheral paresis	3 (10.7%)	1 (16.7%)	0 (0%)	4 (10%)	0.10
	Bleeding	0 (0%)	1 (16.7%)	0 (0%)	1 (2.5%)	
	Paraparesis	0 (0%)	1 (16.7%)	0 (0%)	1 (2.5%)	
	Multiorgan failure	1 (3.6%)	0 (0%)	0 (0%)	1 (2.5%)	
	Cerebral ischemia	5 (17.9%)	0 (0%)	0 (0%)	5 (12.5%)	
Hospitalization time (days)		20 (15–28)	22 (11–30)	21 (19–23)	21 (15–29)	0.91
Next-stage stent graft implantation		4 (14.3%)	2 (33.3%)	0 (0%)	6 (15%)	0.27

AAD—acute aortic dissection; FET—frozen elephant trunk.

**Table 4:** Intraoperative characteristics of consecutive groups of patients: *—post hoc statistically significant group 1 vs. group 2 comparison; +—post hoc statistically significant group 1 vs. group 3 comparison; †—post hoc statistically significant group 1 vs. group 4 comparison; •—post hoc statistically significant group 2 vs. group 4 comparison.

Consecutive Number of Patients	1–10	11–20	21–30	31–40	Total	*p*
Hypothermia temperature (°C)	28 (25–28)	29 (28–32)	30 (28–32)	28 (28–32)	28 (28–32)	0.12
Surgery time (min)	533 (500–560)	405 (280–470)	320 (300–385)	256 (235–285)	355 (280–488)	<0.001 +†•
Cardiopulmonary bypass time (min)	327 (299–360)	206 (150–243)	199 (172–231)	160 (157–172)	206 (160–301)	<0.001 *+†
Aortic cross-clamp time (min)	165 (115–208)	125 (90–140)	122 (118–135)	110 (101–115)	120 (111–155)	0.03 †
Distal hypothermic circulatory arrest time (min)	40 (40–50)	30 (10–35)	43 (30–43)	30 (25–30)	35 (30–43)	0.04

AAD—acute aortic dissection; FET—frozen elephant trunk.

## Data Availability

Data are available from the corresponding author upon reasonable request.

## Supplementary Materials

The following supporting information can be downloaded at https://www.mdpi.com/article/10.3390/jcm13216544/s1, Table S1. Preoperative characteristics of consecutive groups of patients. Table S2. Postoperative characteristics and complication rates of consecutive groups of patients.
