# Supplementary material for: The Frozen Elephant Trunk Procedure—8 Years of Experience from Poland
Source: J Clin Med. 2024 Oct 31;13(21):6544. doi: 10.3390/jcm13216544 (PMC11547153; doi:10.3390/jcm13216544)
Supplement: Supplementary file 1 [file jcm-13-06544-s001.zip › jcm-3274010-supplementary.pdf]

Supplementary Table 1. Preoperative characteristics of consecutive group of patients.

Consecutive number of patients		1-10	11-20	21-30	31-40	general population	p
Age (years)		66 (55 - 71)	60 (56 - 65)	53 (48 - 64)	62 (42 - 63)	60 (53 - 66)	0.37
Sex		5 (50%)	7 (70%)	9 (90%)	6 (60%)	27 (67.5%)	0.26
Body mass index (kg/m2)		25.2 (22.2 - 28.1)	30.7 (26 - 33.5)	23.9 (22.4 - 29.4)	29.4 (27.2 - 32.4)	27.8 (23.4 - 30.7)	0.09
DM2		0 (0%)	1 (10%)	1 (10%)	1 (10%)	3 (7.5%)	0.78
Hypertension		10 (100%)	8 (80%)	10 (100%)	9 (90%)	37 (92.5%)	0.27
Atrial fibrillation		0 (0%)	1 (10%)	1 (10%)	0 (0%)	2 (5%)	0.55
Chronic heart failure		0 (0%)	1 (10%)	0 (0%)	0 (0%)	1 (2.5%)	0.38
Pulmonary disease		0 (0%)	6 (60%)	2 (20%)	3 (30%)	11 (27.5%)	0.02
Ever smoker	active	2 (20%)	7 (70%)	7 (70%)	6 (60%)	22 (55%)	0.24
	previous	7 (70%)	3 (30%)	2 (20%)	3 (30%)	15 (37.5%)	
Vascular disease	Peripheral	1 (10%)	6 (60%)	3 (30%)	3 (30%)	13 (32.5%)	0.13
	cerebral	1 (10%)	0 (0%)	0 (0%)	0 (0%)	1 (2.5%)	
	both	1 (10%)	2 (20%)	0 (0%)	0 (0%)	3 (7.5%)	
Non-elective surgery		8 (80%)	8 (80%)	8 (80%)	7 (70%)	31 (77.5%)	0.93
Tamponade		1 (10%)	3 (30%)	1 (10%)	2 (20%)	7 (17.5%)	0.59
Ejection Fraction (%)		43 (40 - 60)	48 (40 - 55)	40 (40 - 50)	40 (35 - 45)	40 (40 - 53)	0.16
Euroscore II		32.5 (24-48.1)	23.5 (15.5-38.7)	20.6 (13.8-29.5)	22.4 (15.6-48.6)	25.9 (11.7 - 38.7)	0.36

Parts of aorta involved	Type A	1 (10%)	2 (20%)	4 (40%)	1 (10%)	8 (20%)	0.15
	Ascending + arch	3 (30%)	4 (40%)	3 (30%)	7 (70%)	17 (42.5%)	
	Ascending + arch + descending	1 (10%)	1 (10%)	1 (10%)	2 (20%)	5 (12.5%)	
	arch + descending	1 (10%)	2 (20%)	2 (20%)	0 (0%)	5 (12.5%)	
	Thoracic+ abdominal	4 (40%)	1 (10%)	0 (0%)	0 (0%)	5 (12.5%)	
Preoperative new onset neurological symptoms	TIA	3 (30%)	2 (20%)	3 (30%)	4 (40%)	12 (30%)	0.90
	peripheral paresis	1 (10%)	1 (10%)	1 (10%)	0 (0%)	3 (7.5%)	
	Cerebral ischemia	0 (0%)	1 (10%)	1 (10%)	2 (20%)	4 (10%)	
Preoperative Ischemic non-neurological symptoms	lower limb ischemia	6 (60%)	1 (10%)	4 (40%)	2 (20%)	13 (32.5%)	**0.01**
	cardiac arrest	3 (30%)	1 (10%)	2 (20%)	0 (0%)	6 (15%)	
	lower limb ischemia and cardiac arrest	0 (0%)	3 (30%)	0 (0%)	0 (0%)	3 (7.5%)	

	mesenteric ischemia	0 (0%)	0 (0%)	1 (10%)	0 (0%)	1 (2.5%)	
	mesenteric and lower limb ischemia	0 (0%)	0 (0%)	1 (10%)	0 (0%)	1 (2.5%)	
previous aortic procedures	AVR	0 (0%)	1 (10%)	1 (10%)	0 (0%)	2 (5%)	0.67
	Stentgraft	2 (20%)	2 (20%)	0 (0%)	0 (0%)	4 (10%)	
	AAD surgery	0 (0%)	1 (10%)	1 (10%)	1 (10%)	3 (7.5%)	
	Bentall procedure	0 (0%)	0 (0%)	1 (10%)	1 (10%)	2 (5%)	

Supplementary Table 2. Postoperative characteristics and complications rate of consecutive group of patients. AAD – acute aortic dissection, FET – frozen elephant trunk.

Consecutive number of patients		1-10	11-20	21-30	31-40	general population	p
30 day mortality		0 (0%)	3 (30%)	0 (0%)	0 (0%)	3 (7.5%)	0.02
complications	peripheral paresis	2 (20%)	0 (0%)	1 (10%)	1 (10%)	4 (10%)	0.14
	bleeding	1 (10%)	0 (0%)	0 (0%)	0 (0%)	1 (2.5%)	
	paraparesis	1 (10%)	0 (0%)	0 (0%)	0 (0%)	1 (2.5%)	
	multiorgan failure	1 (10%)	0 (0%)	0 (0%)	0 (0%)	1 (2.5%)	

	cerebral ischemia	0 (0%)	4 (40%)	0 (0%)	1 (10%)	5 (12.5%)	
hospitalization time (days)		31 (19 - 52)	14 (10 - 23)	21 (17 - 27)	19 (16 - 22)	21 (15 - 29)	**0.049**
next stage stentgraft implantation		3 (30%)	1 (10%)	2 (20%)	0 (0%)	6 (15%)	0.27

AAD – acute aortic dissection, AVR – aortic valve replacement, DM2- diabetes mellitus type 2, FET – frozen elephant trunk, TIA – transient ischemic attack.
